# Diagnostic Precision in Lyme borreliosis: Assessing VlsE and C6 Antigens in a Pediatric Cohort

**DOI:** 10.3390/diagnostics13233547

**Published:** 2023-11-28

**Authors:** Marta Wozinska, Kacper Toczylowski, Dawid Lewandowski, Ewa Bojkiewicz, Robert Milewski, Artur Sulik

**Affiliations:** 1 Department of Pediatric Infectious Diseases, Medical University of Bialystok, 15-274 Bialystok, Polandkacper.toczylowski@umb.edu.pl (K.T.);; 2 Department of Biostatistics and Medical Informatics, Medical University of Bialystok, 15-295 Bialystok, Poland; robert.milewski@umb.edu.pl

**Keywords:** Lyme borreliosis, *Borrelia burgdorferi*, pediatric diagnosis, recombinant antigens, prevention, diagnostic precision

## Abstract

(1) Background: Lyme borreliosis (LB) is a tick-borne disease known for its diagnostic challenges. Conventional two-tiered testing (CTTT) for antibodies is time-consuming, has low sensitivity in the early stages of disease, and sometimes generates false-positive IgM immunoblots. To tackle this issue, modified two-tiered testing (MTTT) was introduced, incorporating recombinant VlsE and C6 antigens to enhance diagnostic accuracy. (2) Methods: In this prospective study, we enrolled children exhibiting symptoms indicative of LB. We collected serum samples at various intervals and subjected them to analysis using standard enzyme immunoassays. We then compared these results with the outcomes from the VlsE and C6 assays. (3) Results: In our study, all 33 patients displaying erythema migrans (EM), a characteristic symptom of LB, exhibited positive responses to the C6 antigen. This finding underscores the potential utility of the C6 antigen as a reliable diagnostic tool for LB. Additionally, we observed a significant reduction in anti-VlsE antibody levels following antibiotic treatment in EM patients. (4) Conclusions: The utilization of recombinant VlsE and C6 antigens in LB diagnostics and monitoring has yielded promising results. Nonetheless, it is imperative for clinicians to exercise caution and interpret results in conjunction with clinical findings, considering the dynamic nature of medical guidelines. Even with recombinant antigen tests, some children with EM tested negative, highlighting the importance of clinical diagnosis for treatment decisions. Furthermore, clinicians should be mindful of the possibility of persistently positive VlsE/C6 test results during LB treatment monitoring.

## 1. Introduction

Lyme borreliosis (LB) is a tick-borne illness caused by the bacteria *Borrelia burgdorferi* sensu lato (s.l) complex [1,2,3]. This complex includes several genospecies, such as *B. garinii*, *B. afzelii*, and *B. burgdorferi* sensu stricto (s.s), which are prevalent in different parts of the world [3,4]. The multitude of clinical symptoms in both adults and children is related to the heterogeneity of *B. burgdorferi* [5,6,7].

LB is transmitted through the bite of an infected tick [8,9]. The distribution of tick bites varies between adults and children, with children being more likely to experience bites on their head and neck [8]. This poses a significant challenge because ticks can be hard to detect in these areas, especially on the scalp. Unfortunately, these areas are strongly associated with a heightened risk of Lyme neuroborreliosis (LNB) development in children [10]. Moreover, the presence of erythema migrans (EM), which is a characteristic rash associated with LB, in the hair may be overlooked [9].

Conventional two-tiered testing (CTTT) is often used to determine the level of antibodies in the blood [11]. This involves performing a first-tier tests that often utilize peptides, recombinant proteins, or whole-cell sonicate preparations (WCS) derived from *Borrelia*, and frequently employ enzyme immunoassay (EIA) techniques, followed by a supplemental immunoblot (Western blot—WB).

The sensitivity of EIA may be lower in the early stages of the disease and may result in false-negative results during this time [12,13]. In addition, WB is a more specific test, but it can be time-consuming and expensive and has a lower sensitivity compared to EIA. It may also give false-negative results during the first weeks of infection, and its interpretation using specific criteria can be challenging [14,15]. Another disadvantage of the serological diagnosis of LB is the persistence of specific IgM and IgG antibodies, despite the use of effective treatment [16].

Due to the limitations of CTTT, modified two-tiered testing (MTTT) has been proposed. MTTT uses two different sequential EIAs without the inclusion of WB but with the use of recombinant proteins or synthetic peptides unique to *Borrelia burgdorferi*, such as the surface lipoprotein E (VlsE) antigen [17,18]. This can lead to minimal cross-reactivity with foreign antigens and can improve the specificity of the test [17,19]. Moreover, the Food and Drug Administration (FDA) approved the use of EIA as a confirmatory test, making immunoblots optional and incorporating enzyme immunoassays into a two-tiered testing approach [20].

The ability of *Borrelia burgdorferi* to induce illness relies heavily on its capability to avoid detection by the immune system when infecting mammals. A known strategy employed by *B. burgdorferi* to evade the immune response involves altering the antigenic properties of a protein known as the lipoprotein VlsE, which shares similarities with the variable major protein (VMP)-like sequence [21]. Among the known antigens, VlsE stands out as the sole antigen that exhibits ongoing variation of its surface epitopes. It consists of six variable regions (VRs) and six low variability invariant regions (IR) [22]. It is believed that recombination in the VRs can impede an effective response from the host immune system, leading to the development of a chronic infection and the survival of spirochetes in humans [23]. Among the six IRs of VlsE, IR_6_, also known as the C6 region, is commonly targeted by the antibody response in infected individuals [24]. The detection of anti-C6 antibodies may be helpful in the serologic diagnosis of LB in humans [25,26,27]. Studies report that tests utilizing the VlsE and C6 peptides show higher sensitivity compared to CTTT [25,28]. Moreover, anti-VlsE and anti-C6 antibodies exhibit a dynamic response to infection, swiftly appearing in the patient’s serum shortly after infection, and typically diminishing following successful treatment [29,30].

Although the utilization of highly specific antigens in the diagnosis and treatment monitoring of LB in adults has shown promise [31], there remains a notable gap in the available data regarding the application of these antigens within the pediatric population. In light of Poland being endemic to LB and considering the unique aspects of LB in children, it is of paramount importance to conduct comprehensive studies tailored to this specific demographic. Our study thus aims to address this gap by assessing the diagnostic and treatment monitoring potential of VlsE and C6 tests in early-stage LB within the pediatric population, focusing on erythema migrans and neuroborreliosis. Furthermore, we aim to investigate whether children initially diagnosed with erythema migrans by family doctors, who are later referred to specialists in infectious diseases, exhibit positive antibody responses. This aspect of our study not only contributes to understanding the effectiveness of these serologic assays but also sheds light on the diagnostic outcomes for children who initially seek care from general practitioners.

## 2. Materials and Methods

### 2.1. Study Design and Setting

In this prospective study, we recruited children who displayed symptoms suggestive of Lyme borreliosis. The study included children under 19 years of age, and enrollment took place between October 2019 and September 2022. The Medical University of Bialystok Children’s Clinical Hospital, Poland, served as the study setting, providing a suitable environment for the evaluation and management of pediatric cases. We aimed to collect serums samples at the time of consultation and then 3, 6, and 12 months later to monitor the treatment of LB.

Only children with symptoms of early LB, i.e., EM and Lyme neuroborreliosis (LNB), were included. The diagnosis of EM in the enrolled children followed the gold standard criteria established for EM diagnosis. The characteristic EM lesions observed exhibited a round, erythematous appearance and could present with warmth and rare instances of pain or itchiness. These lesions gradually expanded over several days, often exceeding 5 cm in diameter. In some cases, central clearing occurred as the rash enlarged, occasionally resulting in a bull’s-eye pattern. These defined criteria were crucial in ensuring accurate and consistent identification of EM cases among the study participants [32,33,34,35]. The history of a skin lesion meeting the criteria for EM diagnosis, along with the description provided by the parents or a photograph of the skin lesion, was used to confirm the presence of EM in these children. This approach ensured that the diagnosis of EM is consistent and accurate based on the established standards.

The diagnosis of LNB was made according to the Polish Society of Infectious Diseases 2015 guidelines [36]. The following criteria were used: (I) neurological symptoms suggestive of LNB (with other causes excluded), (II) CSF pleocytosis, and (III) specific antibodies in the CSF (produced intrathecally). Patients with positive serology, but other manifestations of LB and non-specific symptoms such as fatigue, headaches, muscle pain, and recurrent fevers were excluded from the study.

### 2.2. Assays

Standard LB EIAs were carried out in the Department of Pediatric Laboratory Diagnostics of the recruiting setting. Borrelia IgM and IgG ELISA Recombinant Antigen assays were used (Biomedica Medizinprodukte GmbH & Co KG, Divischgasse, Wien, Austria). In the IgM class, p21 OspC (*B. afzelii*), p21 OspC (*B. garinii*), p41/l (*B. bavariensis*), and VlsE (fusion protein of different *Borrelia* genospecies) were used. In the IgG class, p21 OspC antigens (*B. burgdorfei* s.s., *B. garinii*), p18 (*B. afzelii*), p100 (*B. afzelii*), and VlsE (fusion protein of different *Borrelia* genospecies) were used. The results were interpreted according to the manufacturer’s instructions and cut-off points: ≥11 Biomedica *Borrelia* units (BBU)/mL (positive), 6 to 11 BBU/mL (equivocal), and <6 BBU/mL (negative).

Standard LB EIAs were compared with the Immunetics C6 Lyme ELISA Kit (Immunetics, Norwood, MA, USA), with them detecting both IgM and IgG antibodies. The results were interpreted according to the manufacturer’s instructions and cut-off points: ≥1.1 Lyme Index LI (positive), 0.91 to 1.09 (LI) (equivocal), and ≤0.9 LI (negative). The specific anti-VLsE antibodies were detected using anti-*B. burgdorferi* VIsE ELISA IgG assays (Euroimmun, Seekamp, Luebeck, Germany), according to the instructions provided by the manufacturer. The results were interpreted according to the manufacturer’s instructions and cut-off points: ≥22 relative units (RU)/mL (positive), 16 to 22 RU/mL (equivocal), and ≤16 RU/mL (negative).

### 2.3. Statistical Analysis

First, we assessed the percent agreement between the standard EIA and EIA with the C6 antigen results. Next, using the Jonckheere–Terpstra test, we performed a trend analysis for the decline in anti-VlsE antibodies after appropriate treatment at 3-, 6-, and 12-month intervals. All analyses were carried out using TIBCO Software Inc. (2017) Statistica, version 13 (Palo Alto, CA, USA), GraphPad Prism version 9.4.0 for Windows (GraphPad Software, San Diego, CA, USA), and R Project version 4.2.2 (The R Foundation for Statistical Computing, Basel, Switzerland).

### 2.4. Ethical Considerations

This research was carried out with the consent of the Bioethics Committee of the Medical University of Bialystok (approval number R-I-002/489/2019).

## 3. Results

### 3.1. Patient Characteristics

A total of 354 children were screened for study inclusion. Two-hundred and eighty four patients were excluded from the analysis because of: unconfirmed LB (*n* = 108), nonspecific symptoms (*n* = 106), missing clinical information (*n* = 24), and a lack of follow-up examinations (*n* = 39). Furthermore, we excluded five Lyme arthritis and two Lyme carditis patients from our study due to their small numbers and the fact that they were unable to return for follow-up at 3, 6, or 12 months because of the COVID-19 pandemic.

After applying the exclusion criteria, a total of 70 children were included in the analysis: 49 children diagnosed with EM and 21 children diagnosed with LNB. The median age of the patients was 8 years, and 43 (61%) of them were female. The age range of the patients varied from a minimum of 12 months (1 year) to a maximum of 18 years.

All of the EM cases were clinically diagnosed according to the guidelines. Children diagnosed with LNB were hospitalized due to symptoms such as meningitis and/or facial nerve palsy. Among these cases, six children exhibited isolated facial nerve palsy, five had lymphocytic meningitis along with facial nerve palsy, and ten children presented with meningitis without facial nerve involvement. For the majority, these symptoms developed within a week before their hospitalization. Remarkably, six children diagnosed with LNB (29%) were admitted to the hospital one month after the initial onset of neuroborreliosis symptoms. Importantly, all LNB patients tested positive for specific anti-*Borrelia burgdorferi* antibodies in both enzyme immunoassays and confirmatory Western blotting.

Unfortunately, our ability to comprehensively assess the diagnostic efficacy of the EIA test with C6 in the entire study group was hindered due to the discontinuation of C6 test production by the manufacturer. Consequently, 16 patients with erythema migrans (EM) and 6 with Lyme neuroborreliosis (LNB) were not tested with the C6 antigen.

Despite the unavailability of the C6 test, we successfully identified the presence of anti-C6 antibodies in a subgroup of 48 children, which included 33 with EM (Table 1) and 15 with LNB (Table 2). The LNB subgroup consisted of four children with facial nerve palsy along with meningitis, five with facial nerve palsy but without meningitis, and six patients with meningitis only.

Notably, almost half of the children with EM who were tested with both C6 and VlsE assays were recruited approximately one month after the appearance of the skin lesion (15/33, 45%). A significant proportion of these children had already received treatment from their family doctors before study recruitment (29/33, 88%, Table 1).

Among the LNB subgroup tested with C6, 40% (6 out of 15) were hospitalized one month after the onset of neuroborreliosis symptoms, with meningitis being the most common clinical presentation. In terms of treatment, the majority of these children received a 21-day course of ceftriaxone antibiotic therapy (12/15, 80%) (Table 2).

### 3.2. Evaluating the Diagnostic Efficacy of the Immunetics^®^ C6 Lyme ELISA Assay in Pediatric Lyme Disease Diagnosis

There was no correlation between EIA IgG and C6 and between C6 and EIA IgM. C6 correlated with anti-VLsE IgG well (Spearman’s R = 0.59, *p* < 0.001).

All 33 patients in the EM group had a positive antibody response to the C6 antigen (Table 3). Among them 27/33 (82%) had positive IgM, and 23 (70%) had positive IgG in the standard EIA assays.

A positive antibody response to the C6 antigen was observed in 12 out of 15 (80%) patients diagnosed with LNB. In these cases, positive IgM or IgG responses in the standard EIA assays were found in all 15 patients. Notably, all four (100%) children presenting with both facial nerve palsy and meningitis, as well as all six (100%) patients with meningitis alone, exhibited positive antibody responses to the C6 antigen.

However, only two out of five (40%) children with facial nerve palsy without meningitis displayed positive results in the C6 assays (Table 3). Among these three LNB patients without a positive antibody response to the C6 antigen, all had an equivocal C6 test result. All of these patients underwent a Western blot test to confirm the diagnosis of Lyme borreliosis, and all of them tested positive. This subgroup included one 11-year-old boy and two girls, aged 11 and 14.

### 3.3. Assessment of Anti-VlsE IgG Assays in Treatment Monitoring in Pediatric Lyme Disease

The usefulness of the anti-VlsE IgG assays in treatment monitoring was assessed in a group of 70 patients. Anti-VLsE IgG levels correlated with standard EIA IgG well (Spearman’s R = 0.72; *p* < 0.001).

**Figure 1:**
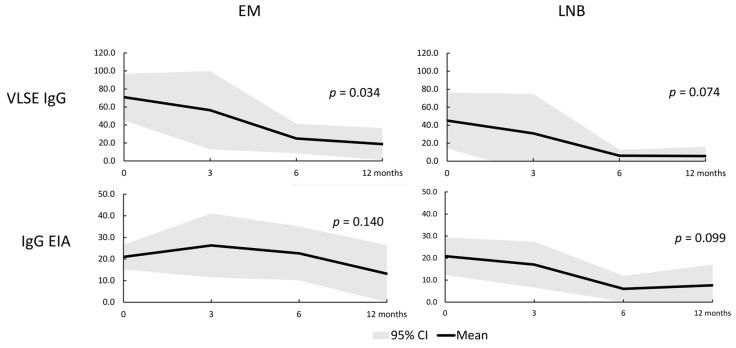
Anti-VlsE and standard Lyme EIA IgG antibody levels in monitoring erythema migrans and Lyme neuroborreliosis treatment.

A positive antibody response to the VlsE antigen was observed in 25/49 (51%) patients diagnosed with EM. Equivocal results were recorded for three (6%) patients. The median VlsE level in the EM group was 27 RU/mL (interquartile range; 8–84 RU/mL). In the EM group, we found a significant decrease in the level of anti-VlsE antibodies after antibiotic treatment after 3, 6, and 12 months from the start of therapy (Figure 1). However, this anti-VlsE antibody titer diminished by a factor of at least four, as suggested by the manufacturer to be the proof of successful treatment, in 13 (50%) patients only.

In the LNB group, 7/21 (33%) tested positive in the VlsE assay, and 1 (5%) had an equivocal result. The median VlsE level was 13 RU/mL (interquartile range; 5–47 RU/mL). There was a non-significant decreasing trend (*p* = 0.07; Figure 1) in the level of anti-VlsE antibodies in this group. After treatment, none of the LNB patients displayed a decrease in antibody titers by a factor equal to or higher than four.

Subsequent tests for patients initially negative for C6 (or VlsE) showed no instances of seroconversion; all negative results remained negative, ambiguous results either remained unchanged or decreased, and the majority of initially positive results exhibited a decline.

The levels of Lyme EIA IgG did not change over time in both EM and LNB groups.

## 4. Discussion

In this study, we assessed the utility of serological tests employing C6 and VlsE antigens for the diagnosis and treatment monitoring of early LB in children. Prior research in the United States has demonstrated that enzyme immunoassays using C6 antigens can enhance the sensitivity of serological LB tests [17,37,38,39]. The usage of the VlsE peptide streamlines the process by requiring only a single test, while traditional whole-cell EIAs necessitate four separate tests. However, it is important to note that the specificity of the VlsE/C6 test may not match that of CTTT [25,40,41]. Furthermore, it is worth considering regional variations in test efficacy. In Europe, for instance, the C6 EIA is sometimes considered less sensitive than traditional EIAs because not all patients exhibit a response to the C6 peptide [42,43,44]. While the IR6 region is conserved, variations in four–five amino acid sequences have been observed among *Borrelia* genospecies [45]. Consequently, the sensitivity of the VlsE/C6 test tends to be higher in the USA, where *B. burgdorferi* sensu stricto predominates as the primary pathogenic species, in contrast to Europe, where there are at least three pathogenic species present. This regional variation may account for the differences in sensitivity observed [3,42,43].

Serological testing is recommended for the diagnosis of LB due to the diverse clinical picture, anatomical differences in the incidence of tick bites in children and missing erythema in the clinical picture [8,9]. Moreover, EM should be differentiated from bacterial cellulitis, eczema, mycosis, and allergic reactions to substances contained in tick saliva [46]. Although serological testing is not advocated for patients with EM, in the case of clinical uncertainties about a rash in LB, serological tests based on EIAs may be performed. In the case of positive and equivocal EIA results, the guidelines recommend WB, which is a time-consuming and expensive procedure that may give a false-negative result in the first weeks of infection [14,34,35]. All of this involves duplicating the visits of small patients, subsequent injections, and collecting serum for re-examination.

The results of our research indicate the usefulness of highly specific antigens in the diagnosis of early LB in children. All patients with EM who had a positive response in the standard EIA were also positive in the C6 antigen test. However, 12 out of 15 patients with LNB had a positive antibody response to the C6 antigen. Therefore, protocols using EIA with C6 could be used for the clinical practice of diagnosing LB in children, especially in patients with a high clinical suspicion of LB (i.e., with tick bites and a rash) [17,47]. Jansson et al.’s research proposed an algorithm with the two-EIA strategy for equivocal cases, where a test with the C6 antigen could be performed after performing a standard EIA on the remaining serum [47]. The authors suggest that if this algorithm had been applied to the 151 sera originally tested using the traditional two-tier strategy, it would have reduced the number of sera requiring WB by 50%, saving reagent costs and staff time. Additionally, it would allow routine laboratories to perform their own testing and confirmation for the majority of their LB patients, reducing turnaround times and the testing burden for reference laboratories, which can then focus on more complex cases [47].

Traditional serological detection of *B. burgdorferi* antibodies, such as EIA and WB, is not recommended for monitoring treatment response or resolving infection because IgG and IgM antibodies can remain elevated for years after infection, despite successful treatment [16,48]. This can lead to repeated unnecessary antibiotic treatment until the serological response is no longer observed. Moreover, for children in endemic areas, previous tick bites might be responsible for positive test results, as the antibodies against *B. burgdorferi* can persist for years even after successful treatment [16,49]. Therefore, a specific marker should be used to assess the effectiveness of LB therapy. The use of specific anti-VlsE antibodies as a marker to assess the effectiveness of LB therapy has been suggested by several researchers [29,30,42,44]. There are several important features that such a marker should possess in order to be effective. It should have high specificity for the pathogen causing LB, should reflect the dynamics of the infection, and should be detectable soon after the infection occurs, allowing for early diagnosis and treatment initiation. Additionally, as the therapy progresses and becomes effective, the marker should decrease in concentration, indicating a positive response to treatment [50]. According to researchers, quantitative measurement of specific anti-VlsE antibodies, fulfills these criteria. These antibodies are highly specific to the pathogen causing LB, and their measurement can indicate the presence and intensity of the infection. Additionally, they have shown potential in reflecting the dynamics of the infection and responding to effective treatment [30,44]. In one of the first publications on this subject, Philipp et al. examined antibody titers against the Vlse/C6 peptide in rhesus monkeys and dogs that were infected with *B. burgdorferi*. The study included seven adult rhesus monkeys, and the researchers observed that there was a rise in anti-C6 IgG antibody titers for 12 weeks after the infection occurred. After the initiation of antibiotic therapy, a decrease in anti-C6 IgG antibody levels was observed in six out of the seven rhesus monkeys. This decrease occurred during a 9-week period of antibiotic therapy and continued until the antibodies completely disappeared 13 weeks after the end of the therapy (specifically, the 34th week after infection) [29]. In 2005, researchers examined the dynamics of anti-VlsE/C6 IgG antibodies in 120 patients with LB, and antibiotic therapy was implemented. Between 4 and 15 months after the treatment, 59.0% of the patients had a total decrease in anti-C6 IgG antibodies and 32.4% of the patients showed at least a four-fold drop in their level [30].

The current study found that there was a statistically significant decrease in the titers of anti-VlsE antibodies among children with EM after effective therapy. In the group of patients with LNB, a decrease in the level of antibodies was also observed after successful therapy, but it was not a statistically significant decrease (*p* = 0.07). In one of the studies also conducted in children with Lyme arthritis, the results revealed that that the decline in antibodies to IR6 does not appear to be useful in assessing Lyme arthritis treatment, as most patients were still positive for IR6 four years after diagnosis [51]. In addition, the results of our study may be influenced by the different clinical course of LB in children and adults. In adults, radicular pain and paresis are more common symptoms, whereas in the pediatric population, facial nerve palsy and subacute meningitis are more frequently observed. Additionally, atypical symptoms such as fatigue, loss of appetite, and mood changes may occur in both children and adults. [52,53]. It is important to be aware of these various clinical features in children with suspected LNB in order to accurately diagnose and manage the disease [32,54]. Generally, the clinical outcome is significantly better in children compared to adults, and long-term neuropsychological disorders are not commonly seen in the pediatric population. In contrast, adults may experience cognitive disorders and persistent or recurrent neurological symptoms [55,56]. Additionally, regarding diagnostic testing, the sensitivity of the test using the VlsE/C6 antigen for neuroborreliosis in children may vary. In Europe, where *B. afzelii* and *B. garinii* are dominant in LNB and in the United States, where *B. burgdorferi* sensu stricto is dominant, the results in monitoring treatment may differ. This suggests that the specific *Borrelia* species present in the region can influence the sensitivity of the test and subsequently impact the diagnosis and monitoring of treatment efficacy [3,57,58].

Most of the previous research on the decline in anti-VlSe/C6 antibody levels primarily focused on adults, and the assessment of the decrease in the level of anti-VlsE/C6 antibodies in children with early LB has not been specifically investigated. It was later recognized that as the infection progresses, the quantitative C6 test used to assess response to treatment becomes less sensitive. Consequently, a decline in VlsE/C6 antibody titers was observed only in successfully treated patients with early localized or early disseminated LB and not in patients with late-stage LB [30]. This observation has clinical implications since information about the decline in VlsE/C6 antibody levels would be more useful in cases of late-stage LB. Late-stage LB refers to cases where the infection has progressed and potentially caused more severe symptoms or complications. However, due to the reduced sensitivity of the C6 test in these cases, assessing treatment response or disease progression based on antibody titers becomes less reliable [30].

It is indeed important to note that while the decline in VlsE/C6 antibodies shows promise, the persistence of positive VlsE/C6 test results does not necessarily equate to an ongoing infection. It is possible for a significant proportion of patients to have persistently positive VlsE/C6 tests even after successful treatment. Therefore, a single VlsE/C6 antibody titer alone is not informative enough to determine the status of the infection after therapy. Instead, it should be considered as part of a longitudinal assessment of the patient’s condition over time [59].

While our study provides valuable insights into the diagnostic potential of serological tests in pediatric Lyme borreliosis, we acknowledge several constraints that warrant consideration. Firstly, the discontinuation of C6 tests by the manufacturer limited our ability to comprehensively assess the diagnostic utility of the EIA test with C6 across the entire study group. This circumstance underscores the need for caution in interpreting the findings related to the C6 assay. Secondly, the ongoing COVID-19 pandemic posed logistical challenges, with parents facing difficulties in attending follow-up visits, thus impacting the completeness of data and reducing the sample size available for analysis. This limitation may influence the generalizability of our results, given the potential selection bias introduced by the constraints of the pandemic. Moreover, the nature of our study design, with follow-up dependent on parental attendance, led to missing data and limited our ability to conduct extensive follow-up examinations. This absence of long-term data may affect the depth of our understanding of the progression and outcomes of LB in the pediatric population. Lastly, the observation that nearly half of the patients with erythema migrans did not exhibit increased VlsE IgG levels poses a limitation on the potential use of this assay in treatment monitoring for this particular subgroup. Acknowledging these limitations is crucial for a nuanced interpretation of our findings, ensuring a balanced understanding of the scope and potential impact of our study.

## 5. Conclusions

The majority of children meeting the clinical criteria for Lyme disease, based on the evident presentation of erythema migrans, received appropriate treatment from family doctors. Nevertheless, these children were referred to specialist clinics for further care. Some children with erythema migrans tested negative for Lyme disease in serologic tests, even when using recombinant antigen-based assays. Clinical diagnosis at this disease stage should remain the gold standard for diagnostics and the basis for treatment initiation. A significant decrease in anti-VlsE antibody titers was observed during erythema migrans treatment, along with a decreasing trend in neuroborreliosis. This suggests the potential use of these antibodies in monitoring Lyme disease treatment in children. In summary, our study underscores the need for optimizing the diagnostic pathway for pediatric Lyme disease, especially in cases of erythema migrans, which can be accurately diagnosed clinically. The challenges posed by serologic tests in detecting Lyme disease in children, despite the use of recombinant antigens, highlight the importance of clinical diagnosis as the primary criterion for treatment initiation. Additionally, our findings suggest that anti-VlsE antibodies may serve as a valuable tool for monitoring the effectiveness of Lyme disease treatment in pediatric patients. These insights contribute to the ongoing efforts to improve diagnosis and care for pediatric Lyme disease cases.

## Figures and Tables

**Table 1:** Demographic and clinical characteristics of children with erythema migrans undergoing C6 and VLsE Lyme ELISA testing.

Patient Number	Age (Years)	Sex	Treatment	Treatment Duration (Days)	Lyme IgG EIA (BBU/mL)	Lyme IgM EIA (BBU/mL)	VlsE (RU/mL)	C6 Lyme ELISA (LI)
1.	9	F	AMX	21	13.1	16.4	20.5	16.1
2.	5	F	AMX	21	11.3	24.1	15.6	13.7
3.	7	F	AMX	14	57.9	4.1	178.0	11.8
4.	15	F	DOX	21	1.8	21.3	4.2	1.6
5.	5	M	AMX	21	10.6	40.2	33.1	100.0
6.	8	M	AMX	21	15.6	41.4	49.1	100.0
7.	12	F	AMX	21	14.7	8.3	74.4	13.6
8.	8	F	AMX	21	4.3	17.5	11.1	15.1
9.	9	F	AMX	21	35.0	20.7	235.0	100.0
10.	3	M	AMX	21	19.6	2.8	49.5	5.7
11.	7	F	AMX	21	11.4	5.6	42.8	12.9
12.	6	F	AMX	21	14.7	38.6	18.6	11.4
13.	4	M	AMX	21	54.8	54.4	250.0	100.0
14.	4	F	AMX	21	7.8	15.2	77.1	100.0
15.	6	M	AMX	21	15.7	11.4	106.3	100.0
16.	11	M	AMX	21	6.8	23.8	4.1	10.8
17.	17	F	AMX	21	1.3	32.1	2.4	4.8
18.	6	F	AMX	21	14.3	12.8	250.0	15.7
19.	5	F	AMX	21	4.7	14.2	11.0	17.6
20.	17	F	AMX	21	13.1	4.0	11.9	20.3
21.	10	M	AMX	21	13.6	51.4	29.5	2.4
22.	9	F	AMX	21	57.0	1.3	5.9	3.3
23.	7	M	AMX	21	3.0	15.6	8.0	9.1
24.	10	F	AMX	21	19.9	60.6	72.6	100.0
25.	3	M	AMX	21	31.9	18.0	204.9	100.0
26.	8	F	AMX	21	19.3	30.3	41.4	23.2
27.	8	F	AMX	21	18.7	60.1	50.0	13.9
28.	6	F	AMX	21	12.8	18.7	5.5	4.5
29.	3	M	CFX	14	12.4	14.7	62.5	100.0
30.	3	F	AMX	21	5.2	83.1	3.9	15.4
31.	7	M	AMX	14	19.2	16.4	15.1	20.3
32.	17	F	DOX	21	42.3	29.9	96.3	13.3
33.	6	F	AMX	28	2.9	17.3	4.6	2.2

F—female; M—male; AMX—amoxicillin; DOX—doxycycline; CFX—ceftriaxone; ELISA—enzyme-linked immunosorbent assays; ELISA ratios: >11 BBU/mL—positive result; 9–11 BBU/mL—equivocal result; <9 BBU/mL—negative result; C6 ratios: ≥1.10 (LI)—positive result; 0.91–1.09 (LI)—equivocal result; ≤0.90 (LI)—negative result; VlsE ratios: ≥22 (RU/mL)—positive result; 16–22 (RU/mL)—equivocal result; ≤16 (RU/mL)—negative result.

**Table 2:** Demographic and clinical characteristics of children with Lyme neuroborreliosis undergoing C6 and VLsE Lyme ELISA testing.

Patient Number	Age (Years)	Sex	Clinical Presentation	Treatment	Treatment Duration (Days)	Lyme IgG EIA (BBU/mL)	Lyme IgM EIA (BBU/mL)	VlsE (RU/mL)	C6 Lyme ELISA (LI)
1.	9	F	FP	CFX	21	7.3	15.4	5.4	1.6
2.	11	M	FP	CFX	21	2.0	15.6	5.2	0.9
3.	11	F	FP	CFX	21	25.6	2.6	18.2	0.9
4.	6	M	FP	CFX	21	47.5	11.2	47.4	15.1
5.	14	F	FP	DOX	21	1.3	30.0	3.7	0.9
6.	17	M	FP + M	DOX	21	66.0	6.0	15.8	4.7
7.	10	M	FP + M	CFX	21	9.0	24	156.0	100.0
8.	15	M	FP + M	CFX	21	7.0	98.5	7.4	16.2
9.	16	F	FP + M	CFX	21	31.0	49.9	27.6	23.5
10.	13	M	M	CFX	21	2.0	200.0	1.5	9.7
11.	4	F	M	CFX	21	9.0	61.0	3.6	7.7
12.	17	F	M	CFX	21	53.4	27.2	217.0	5.6
13.	8	M	M	CFX	21	32.5	10.1	87.6	16.4
14.	15	F	M	CFX	21	3.0	23.9	46.5	100.0
15.	7	F	M	CFX	21	22.6	1.4	4.6	1.2

Abbreviations: F—female; M—male; DOX—doxycycline; CFX—ceftriaxone; ELISA—enzyme-linked immunosorbent assays; FP—facial nerve palsy; M + FP—facial nerve palsy with meningitis; M—meningitis only; ELISA ratios: >11 BBU/mL—positive result; 9–11 BBU/mL—equivocal result; <9 BBU/mL—negative result; C6 ratios: ≥1.10 (LI)—positive result; 0.91–1.09 (LI)—equivocal result; ≤0.90 (LI)—negative result; VlsE ratios: ≥22 (RU/mL)—positive result; 16–22 (RU/mL)—equivocal result; ≤16 (RU/mL)—negative result.

**Table 3:** Percentage agreement between the Immunetics^®^ C6 Lyme ELISA kit, Euroimmun ^®^ VIsE ELISA IgG, and standard *Borrelia* IgM and IgG enzyme immunoassays.

Results	EM *n* (%)	LNB *n* (%)
Positive anti-VlsE	25/49 (51%)	7/21(33%)
Equivocal VlsE	3/49 (6%)	1/21 (5%)
Positive anti-C6	33/33 (100%)	12/15 (80%)
Equivocal C6	0/33 (0%)	3/15 (20%)
Positive IgG EIA	30/49 (61%)	11/21 (52%)
Positive IgM EIA	40/49 (82%)	14/21 (67%)
+IgM EIA and +C6	27/33 (82%)	8/15 (53%)
+IgG EIA and +C6	23/33 (70%)	5/15 (33%)
−IgM EIA and +C6	6/33 (18%)	7/15 (47%)
−IgG EIA and +C6	10/33 (30%)	10/15 (67%)

+ indicates positive test result; − indicates negative test result. Abbreviations: C6, Immunetics^®^ C6 Lyme ELISA; EIA, Euroimmun ^®^ VIsE ELISA IgG standard *Borrelia* enzyme immunoassay; EM, erythema migrans; LNB, Lyme neuroborreliosis.

## Data Availability

The datasets used during the current study are available from the corresponding author upon reasonable request.
